# Supplementary material for: Disposal practices of expired and unused medications among households in Mwanza, Tanzania
Source: PLoS One. 2021 Feb 4;16(2):e0246418. doi: 10.1371/journal.pone.0246418 (PMC7861449; doi:10.1371/journal.pone.0246418)
Supplement: S1 Questionnaire — (DOCX) [file pone.0246418.s002.docx]

### QUESTIONNAIRE IN ENGLISH

| 1.Gender………………………  2. Residence…………………  3. Age……………………  4. Occupation……………………… | 5. Marital status   1. Single 2. Married 3. Widow 4. divorced 5. separated |
| --- | --- |
| 6. Education status   1. illiterate 2. Primary education 3. O- level education 4. A-level education   e) College and university | 7. Do you have any leftover (unused) drugs in your house now?   1. yes 2. no |
| 8. what type of medicine do you have?   \|  \| AVAILABLE \| \| QUANTITY \| \| --- \| --- \| --- \| --- \| \| YES \| NO \| \| ANALEGESIC \|  \|  \|  \| \| ANTIBIOTICS \|  \|  \|  \| \| ANTI INFLAMATORY \|  \|  \|  \| \| ANTI ACID \|  \|  \|  \| \| ANTI HISTAMINE \|  \|  \|  \| \| ANTI COUGH \|  \|  \|  \| \| Other …………. \|  \|  \|  \| | 9. Where do you store them?   1. in 2. On top of a fridge 3. In a cupboard 4. Other ……………………………………   ………………………… |
| 9. Why do you have those left over drugs in your house?  a)I recovered so I decide to stop medication  b)I left them due to intolerable side effects  c) I changed treatment  d) forgetfulness  e) Other mention ……………………………………. | 10. What do you do with those leftover drugs?  a)I give them to neighbor with the same condition  b) I threw them to environment  c) I threw them into trashes  d) I threw them toilet  e) I flash them down the sink.  f) Other mention …………………………  ……………………………………………… |
| 11. Why did you dispose left over(unused) medicines? Because;  a) they were expired  b)I didn’t complete the prescribed full dose  c) I notice change in the medicines.  d) Others ……………………………….  ……………………………… | 12. Where do you throw left out/expired medicines?   1. Dust bin 2. toilet 3. Flash them down the sink 4. Return to the pharmacy, health   Centers or hospitals   1. burn them 2. buried in the ground 3. Other………………………………….. |
| 13. Where did you learn about medication disposal?  a) Never learned  b) Book  c) Seminar  d)media( TV and radio)  e)friends or neighbors | 14. Have you ever heard of any program on disposal of expired drugs?  a) Yes  b) No |
| 15. Have ever asked one who works in medical field about how to properly discard left over medicines  a)yes  b)no | 16. Who did you ask?  a)doctor  b)pharmacist  c)nurse  d)other |
| 17. Do you know that improper discard of medicines is dangerous to human health and surrounding environment?  a)yes  b) no  if yes answer question 17 | 18.how does improper disposal of medicines harm the environment and human health in general?  (mention 3)  ……………………………….. |
| 19. What do you do when you’re not sure about how to discard the left over medicines?  a) Ask doctor  b) Ask the pharmacies  c) I make decision myself  d) Other | 20. Do you place medicines in a sealed bag or container before discarding?  a) Yes  b) No |
| 21 Do you think it necessary that community member should be informed about safe disposal of medication?  a) yes  b)no | 22. Which way did you think will be the best way to educate community about proper disposal of medicines?  a) Pharmaceutical companies should keep the correct step to dispose unused medication on every medication box and cover.  b)government should make campaign and teach the public right way to dispose medication starting from community, school level and also university level  c)advertisement through television, radio and newspaper  d) Other………………………….. |

### QUESTIONNAIRE IN SWAHILI

| 1. Jinsia………………………  2 Umri…………………………  3 Makazi……………………… | 4..Hali yandoa  a) nimeoa/ kuolewa  b) sijaolewa  c)tumetengana  d)tumetalakiana  d)mjane |
| --- | --- |
| 4. elimu  a)sijasoma kabisa  b)elimu ya kidato cha nne  c)elimu ya kidato cha sita  d)elimu ya chuo | 5.Unafanya kazi gani?  a)msaidizi wa kazi za nyumbani  b)mama wanyumbani  c)mfanya biashara  d)mkulima  e)mwajiriwa  f)nyingine taja……………………………………………. |
| 6. unadawa ambazo umebakiza na huzitumii hapa kwako kwa sasa?  a)ndio  b)hapana | \| DAWA \| Upatikanaji \| \| \| QUANTITY \| \| \| --- \| --- \| --- \| --- \| --- \| --- \| \| ndio \| Hapana idadi \| \| \| Dawa za maumivu \|  \|  \|  \| \| \| Dawa ya vidonda tumbo \|  \|  \| \|  \| \| \| Dawa ya kikohozi \|  \|  \| \|  \| \| \| Dawa za kuzuia kuharisha \|  \|  \| \|  \| \| \| Dawa za malaria \|  \|  \| \|  \| \| \| Dawa za macho,pua, sikio \|  \|  \| \|  \| \| \| Nyinginezo \|  \|  \| \|  \| \| |
| 8. unaziifidhi wapi?  a)kwenye kopo  b) juu ya jokofu  c)kwenye kabati  d)sehemu nyingine taja…………………………………………. | 9. .kwanini umebakiza dawa?  a)nilipona  b)madhara ya dawa yalinifanya nisitishe kutumia  c)nilisahau  d)nilibadilishiwa dawa  e)sababu nyingine,  Taja…………………………. |
| 10. Unafanya nini na dawa usizoziitaji?  a) Natupa kwenye taka za nyumbani  b)natupa chooni  c)narudisha kwenye duka la dawa  d)nachoma  e)nachimbia chini  f)nampa mtu atakayehitaji | 11. Ulishawahikutupadawaambazohuzitumii?  a)ndio  b)hapana. |
| 12. Kwanini ulitupa?  a) zilibadilika rangi.  b)nilipona.  c)ziliisha mda wake.  d)sijawahi kutupa huwa namaliza dozi.  e)sababu nyingine taja…………………..  …………………………………… | 13.Huwa.unatupawapidawa  a)kwenye taka zanyumbani  b)natupa chooni  c)narudisha kwenye duka la dawa  d)nachoma  e)nachimbiachini  f)nampa mtu atakayehitaji |
| 14.Umejifunza wapi kuhusu utupaji wa madawa yasiyofaa tena kwa matumizi?  a)vitabu  b)semina  c)vyombo vya habari( redio na runinga)  d)rafiki/ jirani c)sijawahi kujifunza | 15. Unajua kwamba utupaji dawa ovyo unamadhari kwenye mazingira?  a)ndio  b)hapana.  Kama ndio jibu swali la 16 |
| 16. madhara gani unayoyafahamu? Taja 3 kwa binadamu na mazingira kwa ujumla.  ………………………………  ………………………………….  ……………………………………… | 17.Ulishawahi kumuulza mtu yeyote anayehusika na masuala ya afya kuhusu utupaji wa madawa yasiyohitajika?  a)ndio  b) hapana |
| 18. Ulimuuliza nani?  a)muuguzi  b)mfamasia  c)daktari | 19.Je unafikiri ni muhimu kwa jamii kujulishwa kuhusu utupaji salama wa madawa yasiyohitajika?  a) Ndio  b) hapana |
| 20. Je unaona ni njia gani zitumike kuelimisha jamii kuhusu utupaji salama wadawa?  a)Viwanda vya utengenezaji dawa ziambatanishe maelekezo jinsi ya utupaji dawa kwa kila kasha la dawa.  b)Serikali ifanye kampeni kuanzia kwenyejamii, mashuleni na kwenye vyuo, kuhusu utupaji salama wadawa  c)elimu itolewe kupitia vyombo vya habari kama magazeti, redio na runinga  d)nyingine taja………………………… | 21. Je unafunga madawa usiyayahitaji kwenye mfuko kabla hujayatupa?  a)ndio  b)hapana |
| 22. Unafayaje unapokuwa huna uhakika kuhusu utupaji wa madawa yasiyo hitajika  a) na muuliza daktari  b)namuuliza mfamasia  d) namuuliza muuguzi  c) nafanya maamuzi yangu mwenyewe |  |
