# Supplementary material for: Disposal practices of expired and unused medications among households in Mwanza, Tanzania
Source: PLoS One. 2021 Feb 4;16(2):e0246418. doi: 10.1371/journal.pone.0246418 (PMC7861449; doi:10.1371/journal.pone.0246418)
Supplement: Attachment — Submitted filename: REBUTTAL LETTER.docx [file pone.0246418.s003.docx]

**Title:** **Disposal practices of expired and unused medications among households in Mwanza, Tanzania.**

Department of pharmacology, Catholic university of Health and Allied Sciences, Mwanza, Tanzania

Email:carol_maro@yahoo.com

30^th^ September, 2019

**Editor, PLOS ONE**

Dear editors,

Please find the edited manuscript addressing reviewers’/editors comments. The comments have been addressed as shown below.

| No. | **editor’s comment** | **Response** | **Date of response** |
| --- | --- | --- | --- |
| 1 | Please ensure that you refer to Table 1, 2 and 3 in your text as, if accepted, production will need this reference to link the reader to the Table. | All tables have been referred in the text as required | 30- 09-2019 |
| 2 | Please find the specific annotated modifications suggested (31 specific annotations) in the attached PDF version of the manuscript you have submitted to improve the language of the manuscript. Many of the annotated comments are found in the Abstract (page 2), Study area and subjects (page 4), Data collection (page 5) and Discussion (page 8) sections of the manuscript. | Language editing has been done as indicated in the manuscript | 06—6-2019 |
| 3 | please upload your minimal data set as a Supporting Information file, or to a public repository such as Figshare or Dryad. | The suggestion has been taken care | 06-05-2019 |
| 4 | Please also ensure that when you upload your file you include separate captions for your supplementary files at the end of your manuscript. | Separate captions has been added | 06-05-2019 |
|  | Objective; it can be better stated by avoiding unnecessary terms like ‘therefore this study was carried out…’ | Editing has been done as suggested by the reviewer | 16th February 2018 |
|  | Conclusion in the abstract and the main manuscript seem different, please revisit | The conclusion has now been modified to match in the abstract and the main manuscript | 16th February 2018 |
|  | The background provides a good context on the issue, however, the rationale for the conduct of the current study has not been provided in a convincing manner. | The rationale has been re-written as suggested by the reviewer | 16th February 2018 |
|  | In addition, no studies showing the extent of disposal of drugs from households were presented in figures from studies internationally or regionally. | Information/data from other studies in the region has now been included | 16th February 2018 |
|  | Similarly, the presence or absence of studies on disposal practice of medicines in Tanzania has not been described, and if available have not been cited. | It has now been made clear that studies on drug disposal practice among households in Tanzania are limited | 16th February 2018 |
|  | In this section no background information about the study area is provided. Adding information on the city and how the geographical area was chosen to conduct the study needs to be provided in the methods part. | The background about the study area has now been provided | 16th February 2018 |
|  | Page 5, line 120; how was sample size determination done? Please explain the rationale behind the number 359. | Detailed information sample size determination has been provided as requested by the reviewer | 16th February 2018 |
|  | Page 5, line 123; Local street leaders? Please provide some context about who these individuals are in relation to reliability of sampling technique. | Local street leaders have been described and their role in sampling is now provided | 16th February 2018 |
|  | Page 6, line 132; questionnaires? Is it prepared by the authors or adopted? Any attempts at validation of the questionnaire? Please describe the parts and overall content of the questionnaire. | Details on the questionnaire has been provided as suggested by the reviewers | 16th February 2018 |
|  | In the methods part, there is no mention of when the study was conducted; please add information on that. | The information on the study duration has been provided | 16th February 2018 |
|  | In all the tables, the % signs repeated in each figure can be avoided by just showing it in the column title cell (e.g. frequency (%), then all the signs in the rows below can be removed for better presentation) | % signs have been removed | 16th February 2018 |
|  | Table 1; please give a footnote describing what O-level and A-level education stands for | the term O level has now been replaced with secondary school and A level has been replaced with High School | 16th February 2018 |
|  | Table 1; if the term ‘businessman’ includes women also in your study, please change to an inclusive term, like ‘business person’ or ‘merchant’ | The word business man has now been replaced with business person | 16th February 2018 |
|  | In table 2 please modify descriptions as; On top of the fridge --> On top of a fridge On the table --> On a table In the handbag --> In a handbag In the box --> In a box | Editing has been done as suggested by the reviewer | 16th February 2018 |
|  | Table 3: presents the relationship between the respondents and drug disposal practice; however, the title of the paper and the objectives indicate to assessing the disposal practice at household level. So the role of information gathered from respondents is supposed to pertain to the household, otherwise, there is no point in presenting the findings as a household character rather than individual behavior. | It has been made clear that the findings represent household practices and not individual practices. | 16th February 2018 |
|  | Page 10, line 224; information about people being not aware of proper disposing ways was not first presented in the results part, it just appears in the discussion for the first time in the manuscript. Presenting the information in the results part first and discussing it in the discussion section would make the argument better. | The possible explanation has been provided in the discussion | 16th February 2018 |
|  | The conclusion tries to generalize the finding to the whole of Tanzania. However the study was limited to Mwanza, please consider revisiting the conclusion to restrict its scope to the area of the study. | the word Tanzania has been replaced with Mwanza | 16th February 2018 |
|  | General comment The manuscripts could be improved by a general revision to improve its English | Revision has been done | 16th February 2018 |
|  | Despite the fact that the topic of this manuscript is interesting and there is a lack in the literature about this important issue in African countries, | The comment has been addressed in response to reviewer 1. Studies from the region describing prescribing practice have now been added | 16th February 2018 |
|  | Methodology is not transparent and iquite clear. Also, results were not adequately presented. | The methodology and Results section have been modified as seen in the response to reviewer 1 comments | 16th February 2018 |
|  | Demographic characteristics are presented in two tables (Table 1 should be excluded). Table 4 is not clear. | Only on table is presenting demographic characteristics. Table 1 has been omitted | 16th February 2018 |
|  | Semi-structured questionnaire should be described in details in order to understand the results section. | The details on the questionnaire has now been provided as requested by both reviewers | 16th February 2018 |
|  |  |  |  |

I would like to thank the reviewers and the editors for their valuable comments which have made this manuscript better than before.

Please contact me if any further clarification is needed.

Sincerely yours,

Karol J. Marwa

**Corresponding author.**
