# Supplementary material for: Disposal practices of expired and unused medications among households in Mwanza, Tanzania
Source: PLoS One. 2021 Feb 4;16(2):e0246418. doi: 10.1371/journal.pone.0246418 (PMC7861449; doi:10.1371/journal.pone.0246418)
Supplement: Attachment — Submitted filename: Reviewer # 1 comments 2019.docx [file pone.0246418.s004.docx]

Reviewer #1: This is a well-conceived study that provides an important assessment of disposal practices of expired and unused medications among households in Mwanza, Tanzania.

**INTRODUCTION**

1. Page 3 lines 59-60: In Tanzania, the only existing guidelines established by TFDA are for disposal of expired or unused medications at community pharmacies, health centers and hospitals.

**The name of the National Medicines Regulatory Authority in Tanzania has been changed from the Tanzania Food and Drug Authority (TFDA) to the Tanzania Medicine and Medical Devices Authority (TMDA). Make the necessary correction.**

**With respect to lines 59-60, consider referencing the Tanzania guideline.**

2. Page 3 lines 60-61: Guidelines for evaluating environmental impact of the existing and new medications are also lacking in developing countries like Tanzania.

**Have you checked with the Environment Protection Agency of Tanzania to see whether they have Environmental Impact Assessment (EIA) guideline in this regard?**

3. Page 3 lines 68-70: This practice is uncommon practice in developing countries including Tanzania due to nonexistence of suitable technology and high economic investment required [7].

**Rewrite sentence and ensure practice is not used twice in the same sentence.**

4. Page 3 lines 74-76: Incineration though not realistic at house hold level is regarded as the best disposal practice for medications [3, 6] as advocated by the available international guidelines which in fact themselves are only intended for national authorities neglecting households’ level [8].

**Name some of the international guidelines that are only intended for national authorities and examples of some of these authorities? Provide further clarity on this.**

5. Page 4 lines 86-88: Improper disposal of medications has been documented in some countries in Africa including Kenya, Ethiopia and South Africa the most used method for medication disposal in household being thrashing into garbage and flushing into toilets[19] [20, 21].

**Consider including studies done in Tanzania.**

**METHODS**

1. **Page 5 lines 102-103:** Kish Leslie (1965)[23] formula was used to calculate the sample size using an estimated population proportion of 0.13 from a similar study done in Ethiopia[24] to obtain a minimum sample of size 174.

**Why was sample size not calculated based on similar studies done in Tanzania? Example given (Baltazary, 2013).**

2. Page 5 lines 106--111: Serial sampling method was employed in choosing households from the list given by Local Street leaders where by every 1st, 3rd and 5th households were picked and visited. In each of the selected households, only one member was picked randomly for interview in order to avoid duplication of information as per Kish Leslie[25] . Local Street leaders have a list of all household members in their streets as part of the local government system operating in Tanzania thus were considered to be a reliable source in the sampling process.

**Are there not data from a more credible source of statutory statistics organization in Tanzania?**

3. Page 5 lines 112-118:

**What is the Ethical clearance number?**

4. Page 5 lines 119-126:

How was the questionnaire developed? Did you use information from other studies to develop it? If yes, reference those studies where you got the information from. How was the questionnaire pre-tested? Provide more details?

**DISCUSSION**

1. **Ensure that result is discussed chronological as presented in the result**
2. **You have lots of grammatical errors and spelling mistake in the discussion**

**Example given:**

- **Line 177……betwen instead of between**
- **Line 176…..possesion instead of possession**

3. **What were the strengths and limitations of your study?**

GENERAL COMMENT:

**This manuscript could benefit from a professional manuscript editing to improve its grammatical, spelling and intellectual clarity.**
